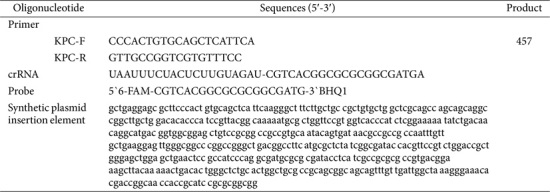# Detection of KPC-Producing Carbapenem-Resistant *Klebsiella pneumoniae* Based on CRISPR Cas12a

**DOI:** 10.4014/jmb.2502.02042

**Published:** 2025-06-12

**Authors:** Qile Gao, Ting Zhang, Yiqun Yuan, Gang Li, Bing Li, Chenglong Xiong

**Affiliations:** 1 Department of Infection Management, Jinshan Hospital of Fudan University, Shanghai 201508, P.R. China; 2 School of Public Health, Fudan University, Shanghai 20032, P.R. China; 3 Department of Laboratory Medicine, Jinshan Hospital of Fudan University, Shanghai 201508, P.R. China; 4 Clinical Medical Research Center, Jinshan Hospital of Fudan University, Shanghai 201508, P.R. China

**Keywords:** Carbapenemase, CRISPR Cas12a, molecular diagnostic, multi-drug resistance, homology

## Abstract

To develop a detection system for *Klebsiella pneumoniae* carbapenemase (KPC) and provide a reference for clinical prevention and control of nosocomial infections caused by multidrug-resistant *K. pneumoniae*. The KPC resistance gene was amplified by PCR. Guided by crRNA, Cas12a specifically identified the resistance gene and activated its trans-cleavage activity. In the detection system, a fluorescence probe was cleaved by activated Cas12a, and the fluorescence signal was measured using a microplate reader. Under optimized conditions, the fluorescence signal appeared within 12 min, peaked at 40 min and completed detection within 60 min. sensitivity: 91.2%, specificity: 84.1%, detection limit: 0.01 ng/μl. The samples were examined by fluorescence-CRISPR Cas12a and PCR. The coincidence rate was 85.9%, *Kappa* value was 0.8. The ROC curve analysis revealed an AUC of 0.916, with an optimal cutoff value of 1.55, sensitivity of 91.2%, and specificity of 84.1%. The CRISPR Cas12a detection of carbapenem-resistant *K. pneumoniae* (CRKP) demonstrates high sensitivity, specificity, and broad applicability. This method requires standard molecular biology equipment but does not rely on sequencing-based platforms.

## Introduction

*Klebsiella pneumoniae* belongs to the family *Enterobacteriaceae*, which includes the well-known genera *Salmonella* and *Escherichia coli* [1]. *K. pneumoniae* is an opportunistic bacterium that can cause serious organ damage and life-threatening diseases [2]. *K. pneumoniae* commonly causes infections in patients who are immunocompromised, have concomitant conditions, or have a preexisting barrier breakdown (such as intravascular devices, endotracheal tubes, and surgical incisions) [3, 4]. In 2017, the World Health Organization included carbapenem-resistant *Enterobacteriaceae* (CRE) in a list of antimicrobial-resistant priority pathogens on which to concentrate future drug development strategies [5]. Of note, CRKP account for 60%–90% of clinical CRE infections in the United States, Europe, and China [6-8], resulting in an increased mortality rate of up to 40%–50%in nosocomial settings [9]. Carbapenem antibacterial drugs have strong effects and widely used, and they are highly stable to extended spectrum β-lactamase (ESBLs) and cephalosporinase. They are the most important antimicrobial drugs for the treatment of multidrug-resistant bacterial infections. Due to the adaptation of bacteria to antimicrobial drugs, the detection rate of CRKP is increasing year by year, bringing great challenges to clinical treatment [10]. *K. pneumoniae* is responsible for up to 10% of nosocomial infections overall [11], and an Italian study also showed that the most frequently identified genes were KPC [12]. A systematic review and meta-analysis made to estimate the prevalence of nosocomial infections due to multidrug-resistant *K. pneumoniae* (MDR-KP) worldwide showed that the prevalence of nosocomial MDR-KP in Europe was estimated at 31.2% (95% CI, 11.5–61.2) [13].

The most important mechanism of intestinal bacterial resistance to carbapenems is carbapenemase production [14]. According to Ambler, carbapenemases are classified into three categories: A, B and D. Class A carbapenemases are most commonly found in the KPC type. Class A enzymes hydrolyze most β-lactam antimicrobial agents, whose activity is partially inhibited by enzyme-containing inhibitors, completely inhibited by boric acid, and not inhibited by ethylenediamine tetraacetic acid (EDTA) [15].

At present, carbapenemase detection methods are divided into phenotype detection and genotype detection. Phenotypic tests mainly include Carbapenemase Cordmann-Poirel Test (CNPT), modified Carbapenem Inactivation Test (mCIM) [16] and Disk Potentiation Test (DPT). Such detection methods can only preliminarily screen carbapenemases and cannot determine the genotype of the tested enzyme. The specific gene detection methods for KPC enzyme mainly include Enzyme Immunochromatography, Quantitative Real-time PCR (qPCR) and Loop-mediated Isothermal Amplification (LAMP) [17, 18]. These methods are fast and efficient. However, special reagents and equipment are needed to detect specific target genes. If the tested gene is different from the target gene, the result will be false negative. KPC mutants often have different drug susceptibility characteristics, and some strains show low levels of carbapenem resistance or even susceptibility, causing difficulties in clinical detection. In conclusion, different methods have their own advantages and disadvantages. The laboratory should choose the appropriate detection method according to its own actual situation, and the routine method can be improved according to its own detection purpose.

**Fig. 1:**
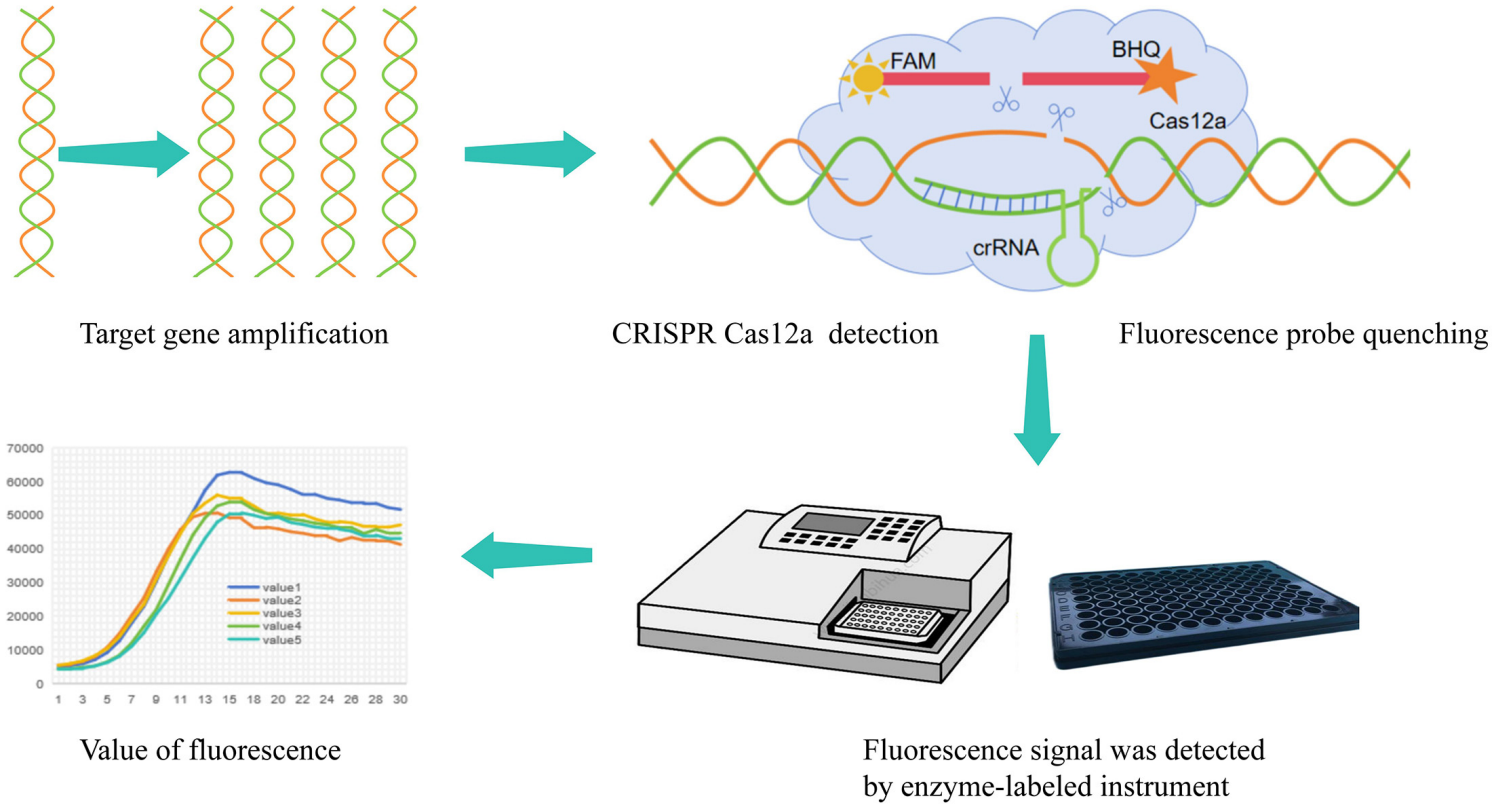
Fluorescence-CRISPR Cas12a assay. First, the target genes were amplified to increase the content of the detected gene.The probe of the target gene was designed with a reporter group labeled at the 5' end and a quench group labeled at the 3' end. The probe was trans-cleavage by CRISPR under the guidance of Cas12a. The reporter and quenched groups were separated to generate a fluorescence signal. Finally, the fluorescence signal was read by the enzyme-labeled instrument.

In recent years, the Clustered Regularly Interspaced Short Palindromic Repeats (CRISPR) has become a powerful gene editing tool [19]. The CRISPR Cas is adaptive acquired immune systems that store the memory of encounters with foreign DNA, primarily that of mobile genetic elements (MGE), in unique spacer sequences derived from MGE and inserted into CRISPR arrays. In 2015, Zhang Feng *et al*. used CRISPR Cas12a for the first time to edit the HEK293FT gene in human cells [20]. At present, CRISPR Cas12a has become an efficient genome editing tool, which is widely used in a large number of organisms, and studies have found that Cas12a has higher specificity than the widely used Cas9 in gene editing and nucleic acid detection [21]. According to CRISPR Cas12a trans-cleavage, a fluorescence detection platform was established to detect KPC gene of carbapenem-resistant *K. pneumoniae*. The amplified target DNA should be added to the CRISPR system. After the Cas12a crRNA complex recognized the target DNA, the trans-cleavage activity was activated, and the double-labeled ssDNA fluorescence quenching groups in the cutting system generated fluorescence signal (Fig. 1). This method has simple operation, low cost, and high sensitivity and specificity.

This article explores the application of CRISPR Cas12a in the detection of KPC, so as to find multi-drug resistant bacteria as early as possible, take control measures as soon as possible, prevent cross-transmission of multi-drug resistant bacteria in the hospital, reduce the incidence of multi-drug resistant bacteria infection in the hospital, and guide the prevention and control of multi-drug resistant bacteria nosocomial infection.

## Materials and Methods

### Reagents and Chemicals

Cas12a and 10×Reaction Buffer were purchased from GenDx (China). Diethyl pyrocarbonate treated water (DEPC), 5×TBE buffer (445 mmol/l Tris; 445 mmol/l Boric acid; 10 mmol/l EDTA), DNA molecular weight Standard Marker (100~2,000 bp), Sterilized ddH_2_O, 6× glycerin gel loading Buffer VII (containing xylene Blue and Bromophenol Blue), SanTaq PCR amplification Kit, Ezup The column bacterial genomic DNA extraction kit were purchased from Shanghai Sangon Biotech Company (China). The gel nucleic acid dye was purchased from Jiangsu keygen Biotech Company (China). The plasmid and crRNA were synthesized and purified by GenScript Biotech Corporation (China). All the primers (Table 1) used in this study were synthesized by Shanghai Sangon Biotech Company. Unless otherwise specified, all water used in this experiment was ultrapure water from Millipore water purification system.

### Methods

**Target DNA Design**

The full-length specific conserved *K. pneumoniae* DNA sequences of the genes encoding KPC enzyme (MT415074) were retrieved from GenBank database at NCBI for designing the most appropriate primers via the Primer explorer V5 software. The crRNA was designed online through http://crispr.mit.edu and off-target effect analysis was performed. The primers were used for PCR and the crRNA was used to guide the CRISPR-Cas12a.

### Standard Recombinant Plasmid Preparations

The target gene was inserted into the MCS region using pUC57 plasmid provided by GenScript Biotech Corporation. The target genes were extended by 16 bp in the upstream and downstream of PCR amplification genes and 489 bp in length. The plasmid synthesis was completed by GenScript Biotech Corporation. The synthesized 4 μg plasmids were diluted with DEPC-treated water to 100 ng/μl, 10 ng/μl, 1 ng/μl, 0.1 ng/μl, 0.01 ng/μl and 0.001 ng/μl, respectively.

### Bacterial Collection and DNA Extraction

After thawing the frozen *K. pneumoniae*, *Acinetobacter baumannii*, *E. coli*, and *Pseudomonas aeruginosa* naturally, they were inoculated into MacConkey Agar and cultured in a constant temperature incubator for 24 h. Select a single colony in LB medium and shake it on a shaker at 36°C for 18 h at 2,000 rpm. Used the Ezup column bacterial genomic DNA extraction kit to extract bacterial genomic DNA according to the following method.

a. Sample processing: Took 1 ml of bacterial solution cultured overnight, added it to a 1.5 ml centrifuge tube, centrifuge at 8,000 rpm for 1 min at room temperature, discarded the supernatant, and collected the bacterial cells. Added 180 μl buffer digest, then added 20 μl Proteinase K solution and shook well. 56°C water bathed for 1 h until the cells were completely lysed.

b. Added 200 μl Buffer BD, thoroughly inverted and mixed, and immersed in a 70°C water bath for 10 min.

c. Added 200μl anhydrous ethanol and thoroughly inverted and mixed.

d. Placed the adsorption column into the collection tube, used a pipette to add all the solution and semi transparent fibrous suspension into the adsorption column, let it stand for 2 min, then centrifuged at 12,000 rpm for 1 min at room temperature, and discarded the waste liquid in the collection tube.

e. Placed the adsorption column in the recovery manifold, added 500 μl PW Solution, centrifuged at 10,000 rpm for 30 sec, and discarded the filtrate.

f. Placed the adsorption column in the recovery manifold, added 500 μl of Wash Solution, centrifuge at 10,000 rpm for 30 sec, and discarded the filtrate.

g. Placed the adsorption column back into the recovery manifold and centrifuged at 12,000 rpm for 2 min at room temperature to remove any remaining Wash Solution.

h. Removed the adsorption column and placed it in a new 1.5 ml centrifuge tube. Added 50-100 μl CE Buffer and let it stand for 3 min. Centrifuged at 12,000 rpm for 2 min at room temperature to collect the DNA solution.

Finally, stored the extracted DNA at -20°C.

DNA had a significant absorption peak at OD_260_ (NanoDrop 2000), the OD_260_/OD_280_ value was 1.7-1.9, and the DNA concentration was more than 200 μg/ml.

### PCR Amplification

Conventional PCR reaction system is composed of 5 μl 10×Taq Buffer with (NH_4_)_2_SO_4_ (750 mM Tris-HCl, 200 mM (NH_4_)_2_SO_4_), 1 μl dNTP Mix , 2 μl forward and reverse primers (10 μM) , 0.5 μl Taq DNA Polymerase, 3 μl MgCl_2_, 2 μl genomic DNA, and ddH_2_O. The final reaction volume was 50 μl. The reaction included denaturation at 95°C for 5 min, followed by 35 cycles of reaction (ie, denaturation at 95°C for 10 sec, annealing at 55°C for 10 sec, extension at 72°C for 30 sec). PCR parameters were referenced to the SanTaq PCR Kit from Sangon Biotech.

### Agarose Gel Electrophoresis

PCR product 5 μl, 6× loading buffer 2.5 μl and gel nucleic acid dye 2.5 μl were blown and mixed repeatedly and added into 1.0% gel, the voltage was set at 110 V, and the setting time was 25 min. After electrophoresis, the gel is imaged in a chemiluminescence imaging system.

### Feasibility of Fluorescence-CRISPR Cas12a Assay

The synthesized 100 ng/μl plasmid 2 μl was used as template DNA for PCR amplification. Group A which all components of the reaction system were added, and group B-D which lacked some components, were explored the feasibility of Fluorescence-CRISPR Cas12a detection method (Table S1).

Fluorescence-CRISPR Cas12a system is composed of 5 μl PCR products, 2 μl 10×Reaction Buffer, 2 μl fluorescent probe (10 μM), 2 μl Cas12a (0.5 μg/μl), 4 μl crRNA (1 μM), and ddH_2_O. The final reaction volume was 100 μl. Detection methods: The reaction mixture system was added into the 96 well enzyme-label plate and put into the enzyme-label instrument for detection. The reaction temperature was 40°C, the fluorescence signal was read every 3 min, the excitation wavelength was 485nm, the emission wavelength was 535 nm, and the binary color mirror was 510 nm [22].

### Evaluation of Fluorescence-CRISPR-Cas12a Assay

**Sample size calculation.** KPC genome amplification: 17 strains of frozen CRKP and 5 strains of non-drug-resistant *Enterococcaceae* bacteria (non-drug-resistant *K. pneumoniae*, *P. aeruginosa*, *Acinetobacter baumannii*, *E. coli*) were amplified by PCR. The PCR products of the above 22 strains were sequenced, and the results showed that 17 strains carried KPC gene and 5 strains did not.

The PCR products of the 22 strains were detected by Fluorescence-CRISPR Cas12a Assay. The results showed that 16 strains carried KPC gene and 6 strains did not carry KPC gene (Table S2).

According to the screening test formula:

$$n={\left[\frac{57.3\times Z_{1-\alpha /2}}{\sin^{-1}\delta /\sqrt{p\left(1-p\right)}}\right]}^{2}$$

α =0.05, δ = 0.1; Calculation sensitivity of pre-experimental test results 94.1%, Reference specificity 97.5%[23], through calculation: n_1_ = 20.02; n_2_ =7.93.

### Definition of Fluorescence-CRISPR Cas12a Assay Results

After removing the interference of background signal, it was judged according to the detection of fluorescence signal.

The formula:

$$F=\frac{F_{C}-F_{N}}{B_{C}-B_{N}}$$

F: Fluorescence intensity at 40 min; B: Fluorescence intensity at 0 min; C: Test results of experimental group; N: negative control; Observing the detection results of positive strains and negative strains, it was found that the fluorescence signal of positive strains had a significant growth trend with the reaction, while the fluorescence signal of negative strains had no significant growth. The detection results could be judged by the difference in the growth trend. According to the related literature CRISPR detection [21], define decision value judging F. F ≥ 2 is positive, F < 2 is negative. Gold standard: PCR amplification of drug resistance gene (KPC), through agarose gel electrophoresis detection, 400-500 bp of high-light bands appeared as drug-resistant strains (CRKP). Compared with gold standard, the sensitivity and specificity of Fluorescence-CRISPR Cas12a assay were calculated. The measurement was repeated and the coincidence rate was calculated.

### Statistical Analysis

Statistical analysis was performed by Statistical Package for Social Sciences (SPSS)version Mac 20.0. Fluorescence signals were reported with descriptive statistics. The normality of distribution of continuous variables was verified with the Kolmogorov-Smirnov test. Continuous variables were expressed as mean ± standard deviation. Statistical comparisons were carried out according to the intention to treat by Student’s *t*-test, Mann-Whitney U test, where appropriate. Receiver operating characteristic curve-ROC was used to represent the sensitivity and specificity pair corresponding to decision threshold of fluorescence signal levels in KPC-Producing carbapenem-resistant *K. pneumoniae* diagnosis. *P* < 0.05 was accepted to be statistically significant.

This study was reviewed and approved by the Medical Ethics Committee of Jinshan Hospital, Fudan University (JIEC 2023-S12).

## Results

### Feasibility of Fluorescence-CRISPR Cas12a Assay

Group A contains the detected components, and group B-D lacks some detected components. The reaction was carried out at 40°C, and the fluorescence signal was read by the enzyme-labeled instrument every 3 min. The reaction time was 60 min, and the fluorescence intensity was plotted according to the fluorescence signal read during the reaction. Group A produced an obvious fluorescence signal, while group B-D did not produce obvious fluorescence signal. The results showed that when the template gene, Cas12a and crRNA were present, the trans-cleavage activity of Cas12a could be activated and the fluorescence signal could be generated (Table S1).

### Detection Threshold of Fluorescence-CRISPR Cas12a Assay

**Fig. 2:**
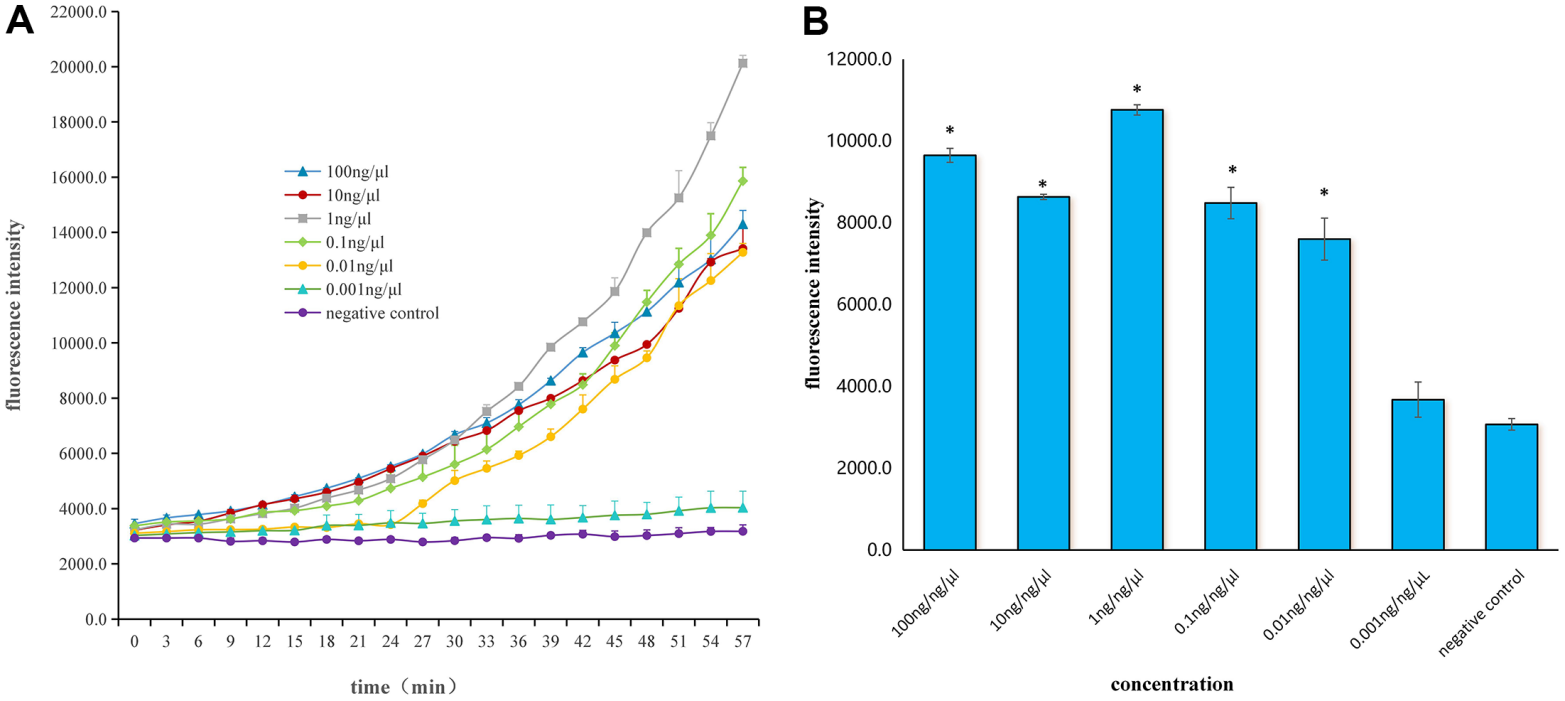
The fluorescence-CRISPR Cas12a assay different concentration plasmid detection curve. (**A**) The plasmids with different concentrations were measured by fluorescent-CRISPR Cas12a assay, and the line chart of 1h average fluorescence intensity was detected. (**B**) The ordinate is the average value of the fluorescence intensity measured at the 42 min, and the abscissa is the plasmid with different concentration. *Indicates a statistically significant difference.

The plasmids were diluted to 100 ng/μl, 10 ng/μl, 1 ng/μl, 0.1 ng/μl, 0.01 ng/μl, 0.001 ng/μl. Different concentrations of plasmids were amplified by PCR. PCR products were detected using the Fluorescent-CRISPR Cas12a Assay. The reaction was carried out at 40°C, and the fluorescence signal was read by the enzyme-labeled instrument every 3 min. The reaction time was 60 min, and the fluorescence intensity was plotted according to the fluorescence signal read during the reaction. The plasmids 100 ng/μl, 10 ng/μl, 1 ng/μl, 0.1 ng/μl, 0.01 ng/μl produced obvious fluorescence signals (Tables S6-S8). The results showed that the threshold value of Fluorescent-CRISPR Cas12a assay was 0.01 μg/μl (Fig. 2).

### Sensitivity and Specificity of Fluorescence-CRISPR Cas12a Assay

**Fig. 3:**
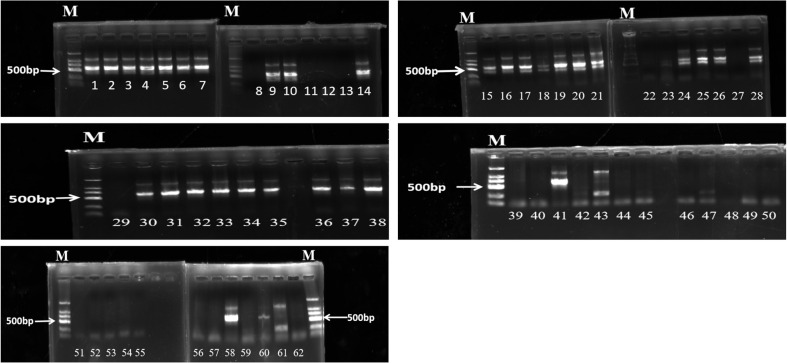
Some target genes were successfully amplified by PCR. The samples (62) were amplified by PCR and detected by agarose gel electrophoresis. M was the standard of DNA molecular weight (100-2,000 bp). Sample number: 8, 11-13, 22-23, 27-29, 39-40, 42-47, 49, 51-57, 59-62 electrophoresis 500 bp did not show bright bands, were negative, the rest were positive.

The 62 samples were detected by PCR (Fig. 3). Agarose gel electrophoresis showed 34 positive and 28 negative. The 62 samples were tested twice by Fluorescent-CRISPR Cas12a assay (Table S3). The results showed that 30 of 34 positive strains were positive and 4 were negative, with a sensitivity of 88.2%; Among the 28 negative strains, 26 were negative and 2 were positive, with a specificity of 92.9% (Table S4).

### Reliability and ROC Curve of Fluorescence-CRISPR Cas12a Assay

**Fig. 4:**
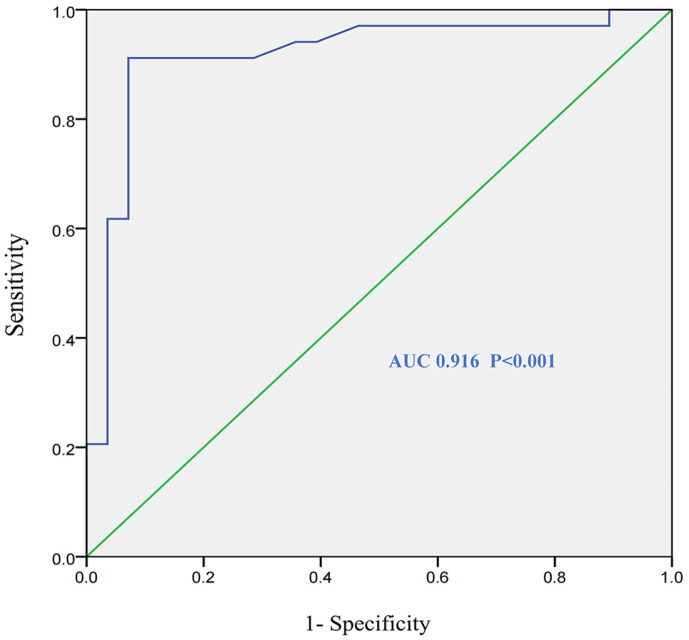
ROC curve of fluorescence-CRISPR Cas12a assay. Clinical samples were tested with Fluorescence-CRISPR Cas12a Assay and PCR. Receiver operating characteristic curve-ROC was used to represent the sensitivity and specificity pair corresponding to test.

The 62 strains of samples were tested by Fluorescence-CRISPRCas12a assay and PCR method (Tables S9-S11). The coincidence rate was 85.9% and *Kappa* value was 0.8 (Table S5). The area under the curve (AUC) formed by the ROC curve and diagonal line was 0.916, the optimal cutoff value was 1.55, the sensitivity was 91.2%, and the specificity was 84.1% (Fig. 4).

## Discussion

At present, the main method of clinical microbial laboratory drug sensitivity test is disk method, which is simple to operate and widely used in clinical practice. However, there are differences between *in vitro* drug susceptibility test and actual bacterial infection, especially between the combination of antimicrobial drugs and *in vitro* drug susceptibility test, and the results of drug susceptibility test are inconsistent with the clinical manifestations and disease changes of patients [24]. Due to individual differences in bacterial species, infection site and amount of bacteria, and the different binding rates of antibiotics to plasma proteins in different populations, pharmacokinetic factors were not considered in the results of *in vitro* drug sensitivity test [25], resulting in inconsistency between *in vitro* drug sensitivity test and actual infection. Therefore, rapid laboratory detection and genotyping of carbapenemase -resistant *Enterobacteria* are essential for the antimicrobial treatment of CRE infection and the prevention and control of nosocomial infection transmission. This study found that the Fluorescence-CRISPR detection has the advantage of rapid detection, and it takes only 40 min for a significant fluorescence signal to appear in the enzyme-labeled instrument.

There are many methods for clinical detection of drug-resistant strains, including disk diffusion method, E-test, micro-broth dilution method and automatic antimicrobial susceptibility detection equipment. Phenotypic detection methods of carbapenemase-resistant strains included CarbaNP, mCIM and eCIM, and β-lactamase inhibitors enhanced antibacterial activity. Genotype detection mainly include PCR, qPCR and other detection methods, and RT-PCR combined with microfluidic technology to identify strains through genotype or gene sequencing analysis [26]. Genotype detection requires standard molecular biology equipment but does not rely on sequencing-based platforms. Genotype detection is not affordable in a normal laboratory. However, PCR results in template-independent primer interactions, resulting in nonspecific products. In particular, primer dimers (PDs) are the product of a double strand formation between two primers. The formation of primer dimers not only reduces the primer concentration in the reaction mixture but also promotes the formation of nonspecific DNA products. This will directly affect the identification of PCR products: non-specific band interference in agarose gel electrophoresis or non-specific SYBR dye embedded in double DNA in real-time fluorescence quantitative PCR [27].To avoid the interference of primer dimers on the experimental results, when designing primers, factors such as primer length, terminal nucleotides of primers, GC content, and Tm value are usually considered, and reaction conditions are optimized. However, due to the exponential amplification of PCR, even if all the above factors are taken into account, non-specific false positive results such as primer dimers will inevitably occur after a long reaction time. This protocol utilizes PCR products complementary to the crRNA sequence to activate CRISPR Cas12a. They have different sequences compared to the specific products and will not activate the trans-cleavage activity of CRISPR Cas12a, thus not generating non-specific fluorescence signals.

For such pathogens, it is urgent to develop more rapid, simple and accurate detection technology to deal with the “bottleneck problem” in the rapid detection of bacteria [28]. Timely and accurate etiological diagnosis is crucial for better early, rapid and accurate antimicrobial treatment information for clinical use, and also provides a reliable basis for nosocomial infection prevention and control. Currently, detection using a combination of PCR and CRISPR can reach a minimum level of 1–10 aM [29]. Gao *et al*. found that the CRISPR Cas12a reaction is performed at a low temperature that is constant without the need for complex temperature control devices. This can be achieved using thermostatic heating units in resource-poor areas. Secondly, the detection time was shorter than those of qPCR methods (>1.5 h) and the 13 respiratory tests commonly used in laboratories (>3 h). Alternatively, the method is sufficient to reliably detect pathogen-derived nucleic acids in most sample types[30]. In this paper, the results show that the sensitivity is 88.2% and the specificity is 92.9%. The fluorescence CRISPR detection method has good sensitivity and specificity and can provide a reliable reference for clinical treatment and prevention and control of hospital infection.

## Conclusion

In this paper, a CRISPR Cas12a based technique is designed and developed in combination with an enzyme-labeler to meet the need for rapid and inexpensive detection of drug-resistant genes. This method allowed us to detect the gene fragment of carbapenemase in the amplified product within 40 min. The new tool has some obvious advantages. First, it requires simple amplification of gene fragments. CRISP Cas12a technology can specifically trans-cleavage fluorescent reporter genes, effectively preventing the influence of primer dimer on the results. Secondly, compared with traditional laboratory detection methods, the method proposed in this topic can greatly shorten the detection cycle, and the operator does not need to undergo repeated training. This new method allows us to accurately detect the genotype of the resistance gene. Finally, a rapid detection platform can be built based on this technology. The platform is suitable for other pathogens commonly found in hospitals, such as methicillin-resistant *S. aureus*, *Clostridium difficile*, and influenza virus. It can be easily detected by simply changing the crRNA. In low-resource areas, the method can still provide accurate detection results. Despite the above advantages of the technology, some limitations of the technology have also been found. For example, CRISPR-based Cas can only detect known genetic sequences, not for unknown pathogens. In addition, current test kits must be stored at −20°C, which is a harsh storage condition. Further optimization is needed to ensure that it can be stored at room temperature for large-scale field screening and clinical differential diagnosis.

## Supplemental Materials

Supplementary data for this paper are available on-line only at http://jmb.or.kr.



## Figures and Tables

**Table 1:** The amplification primer and oligonucleotide sequence in the detection system.