# Supplementary material for: Detection of KPC-Producing Carbapenem-Resistant Klebsiella pneumoniae Based on CRISPR Cas12a
Source: J Microbiol Biotechnol. 2025 Jun 12;35:e2502042. doi: 10.4014/jmb.2502.02042 (PMC12197814; doi:10.4014/jmb.2502.02042)
Supplement: Supplementary file 1 [file jmb-35-e2502042-supple.pdf]

J. Microbiol. Biotechnol. 2025. 35: e2502042
https://doi.org/10.4014/jmb.2502.02042

Supplementary Tables

Table S1. The different components of Fluorescence-CRISPR Cas12a Assay.

	A	B	C	D
Cas12a	+	-	+	+
CrRNA	+	+	-	+
probe	+	+	+	-
template/ amplification product	+	+	+	+

Table S2. The sensitivity and specificity of pre-experiment.

Fluorescence-CRISPR Cas12 Assay	Gold Standard（PCR）	
	positive	negative
positive	16	0
negative	1	5
total	17	5

Table S3. Comparison of fluorescent-CRISPR Cas12a with PCR detection results.

Test No.	PCR	Fluorescence-CRISPR1		Fluorescence-CRISPR2		Test No.	PCR	Fluorescence-CRISPR1		Fluorescence-CRISPR2	
1	+	4.23	+	12.60	+	32	+	3.16	+	12.12	+
2	+	11.03	+	14.06	+	33	+	4.40	+	6.41	+
3	+	6.97	+	17.42	+	34	+	3.28	+	7.96	+
4	+	4.49	+	17.44	+	35	+	2.72	+	6.97	+
5	+	3.03	+	16.39	+	36	+	1.25	-	3.34	+
6	+	4.57	+	18.01	+	37	+	2.88	+	2.59	+
7	+	6.61	+	16.56	+	38	+	2.97	+	5.61	+
8	-	1.08	-	1.16	-	39	-	1.28	-	1.11	-
9	+	4.55	+	17.39	+	40	-	1.22	-	1.07	-
10	+	1.60	-	17.46	+	41	+	4.20	+	1.12	-
11	-	1.09	-	1.23	-	42	-	1.22	-	1.07	-
12	-	1.10	-	1.21	-	43	-	1.33	-	1.14	-
13	-	1.50	-	2.75	+	44	-	1.25	-	1.13	-
14	+	5.26	+	14.61	+	45	-	1.25	-	1.08	-
15	+	4.23	+	14.80	+	46	-	1.19	-	1.09	-
16	+	7.49	+	16.47	+	47	-	1.24	-	1.06	-
17	+	7.22	+	17.83	+	48	+	1.23	-	1.10	-
18	+	9.51	+	18.16	+	49	-	1.19	-	1.10	-
19	+	9.70	+	16.51	+	50	+	1.17	-	1.17	-
20	+	2.86	+	16.51	+	51	-	1.19	-	1.30	-
21	+	12.00	+	13.61	+	52	-	1.18	-	1.25	-
22	-	1.18	-	1.12	-	53	-	3.54	+	1.18	-
23	-	9.68	+	6.31	+	54	-	1.19	-	1.19	-
24	+	11.45	+	9.31	+	55	-	1.29	-	1.18	-
25	+	11.13	+	8.85	+	56	-	1.20	-	1.26	-
26	+	12.20	+	14.21	+	57	-	1.20	-	1.13	-
27	-	1.32	-	1.25	-	58	+	12.31	+	1.43	-
28	+	8.65	+	1.18	-	59	-	1.23	-	1.24	-
29	-	1.22	-	1.33	-	60	-	1.26	-	1.39	-
30	+	2.56	+	12.14	+	61	-	1.22	-	1.28	-
31	+	3.38	+	2.09	+	62	-	1.23	-	1.17	-

Table S4. Sensitivity and specificity of Fluorescent-CRISPR Cas12a assay.

	**Fluorescence-CRISPR Cas12a Assay**	**Gold Standard（PCR）**	
		positive	negative
First detection	positive	30	2
	negative	4	26
	**total**	34	28

Table S5 Reliability and ROC curve of Fluorescence-CRISPR Cas12a Assay

**Second detection**	**First detection**		**total**
	positive	negative	
positive	28	3	31
negative	4	27	30
**total**	32	30	62

Table S6. Fluorescence -CRISPR Cas12a to detect fluorescence intensity of 100ng/μl, 10ng/μl, 1ng/μl, 0.1ng/μl, 0.01ng/μl plasmids (first time).

Time (s)	100 ng/μl	10 ng/μl	1 ng/μl	0.1 ng/μl	0.01 ng/μl	0.001 ng/μl	Negative control
0	3570	3272	3244	3407	3082	2959	2570
180	3746	3446	3464	3520	3140	3019	2644
360	3815	3515	3424	3552	3206	2978	2690
540	3951	3751	3600	3539	3214	3032	2683
720	4185	4155	3687	3685	3228	3071	2719
900	4494	4344	3881	3690	3306	3080	2771
1080	4748	4538	4332	3840	3289	3118	2777
1260	5128	4928	4578	3944	3424	3095	2760
1440	5581	5381	4969	4384	3393	3173	2855
1620	6001	5901	5735	4736	4154	3222	2816
1800	6764	6364	6578	5199	4987	3307	2974
1980	7232	6833	7387	5769	5432	3370	2920
2160	7887	7543	8398	6650	5897	3438	3076
2340	8705	7981	9806	7657	6544	3313	3117
2520	9773	8654	10850	8127	7577	3496	3239
2700	10604	9324	12161	10162	8655	3474	3217
2880	11335	9907	14064	11718	9433	3593	3264
3060	12632	11143	15835	13174	11321	3688	3346
3240	13560	12981	17786	14528	12231	3747	3343
3420	14837	13837	20303	16351	12451	3666	3451

Table S7. Fluorescence -CRISPR Cas12a detected fluorescence intensity of 100ng/μl, 10ng/μl, 1ng/μl, 0.1ng/μl, 0.01ng/μl plasmids (second time).

Time (s)	100 ng/μl	10 ng/μl	1 ng/μl	0.1 ng/μl	0.01 ng/μl	0.001 ng/μl	Negative control
0	3273	3089	3239	3314	3011	3122	2721
180	3541	3315	3413	3488	3182	3142	2765
360	3764	3565	3482	3557	3216	3284	2801
540	3897	3987	3718	3793	3264	3301	2885
720	4076	4076	4122	4197	3315	3345	2895
900	4356	4344	4311	4386	3361	3365	2807
1080	4765	4677	4505	4580	3387	3823	2833
1260	5087	4983	4895	4970	3423	3844	2873
1440	5456	5543	5348	5423	3587	3989	2899
1620	5987	5876	5868	5943	3945	3886	2784
1800	6543	6543	6331	6406	4367	4028	2774
1980	6877	6778	7800	6875	4987	4172	2963
2160	7555	7521	8510	7585	5643	4196	2850
2340	8543	7987	9948	8023	5987	4209	2990
2520	9456	8564	10621	8896	6547	4170	2992
2700	9897	9453	11291	9366	7710	4352	2872
2880	10769	9987	13874	10988	8948	4295	2906
3060	11345	11433	14110	12185	9376	4489	2971
3240	12008	12781	16948	13023	10286	4719	3091
3420	13876	12543	19804	15879	11906	4720	3044

Table S8. **Fluorescence -CRISPR Cas12a Detection of 100ng/μl, 10ng/μl, 1ng/μl, 0.1ng/μl, 0.01ng/μl fluorescence intensity of plasmids (the third time).**

Time (s)	100 ng/μl	10 ng/μl	1 ng/μl	0.1 ng/μl	0.01 ng/μl	0.001 ng/μl	Negative control
0	3527	3306	3217	3420	3109	3002	2643
180	3703	3480	3437	3533	3167	3088	2711
360	3772	3549	3397	3565	3233	3134	2775
540	3908	3785	3573	3552	3241	3141	2812
720	4142	4189	3660	3698	3255	3185	2877
900	4451	4378	3854	3703	3333	3195	2895
1080	4705	4572	4305	3853	3316	3223	2911
1260	5085	4962	4551	3957	3451	3244	2889
1440	5538	5415	4942	4397	3420	3289	2901
1620	5958	5935	5708	4749	4181	3286	2874
1800	6721	6398	6551	5212	5014	3328	2898
1980	7189	6867	7360	5782	5459	3272	2913
2160	7844	7577	8371	6663	5924	3296	2903
2340	8662	8015	9779	7670	6271	3309	2915
2520	9730	8688	10823	8440	7104	3370	2899
2700	10561	9358	12134	10175	8382	3452	2908
2880	11292	9941	14037	11731	9260	3495	2956
3060	12589	11177	15808	13187	10348	3589	2988
3240	13517	13015	17759	14141	11258	3619	2994
3420	14194	13871	20276	15364	12478	3720	3011

Table S9. Fluorescence -CRISPR Cas12a was used to detect fluorescence intensity in 62 samples.

Time (s)	A1	A2	A3	A4	A5	A6	A7	A8	A9	A10	A11	A12	A13	A14	A15	A16	A17	A18	A19	A20	A21	A22
0	3266	3470	3472	3372	3271	3556	3703	3095	3182	3206	3164	3239	3207	3534	3314	3295	4720	4535	4956	3939	4302	3086
180	3460	3698	3811	3699	3401	3717	3944	3157	3225	3369	3079	3226	3329	3764	3467	3544	5002	4882	5209	3999	4554	3184
360	3548	4009	4035	3904	3520	4027	4292	3135	3474	3306	3146	3148	3365	3954	3647	3883	5458	5208	5825	3979	4878	3193
540	3684	4214	4347	4220	3732	4283	4891	3135	3747	3517	3112	3169	3361	4285	3954	4431	6190	6004	6848	4115	5548	3300
720	3726	4485	4932	4659	4011	4749	5545	3213	3977	3471	3020	3111	3430	4741	4222	5163	8092	7732	9024	4176	7503	3341
900	3917	5009	5454	5066	4302	5330	6508	3229	4411	3631	3001	3249	3481	5233	4686	6030	10580	10719	12664	4452	10139	3473
1080	4094	5409	6515	5617	4692	5888	7484	3203	4833	3744	3208	3149	3620	6123	5385	6989	14033	15067	18413	5001	14397	3472
1260	4347	6125	7897	6507	5024	6597	8907	3303	5492	3847	3155	3227	3715	6958	5852	8493	18309	20426	25076	5527	19465	3337
1440	4638	6537	9564	7183	5466	7566	10450	3230	6269	4061	3207	3170	3778	8108	6646	10230	22238	25521	32054	6152	25325	3451
1620	4955	7266	11405	8260	6017	8812	12385	3260	7377	4154	3302	3325	3914	9571	7624	11971	26032	30420	38923	6977	30620	3493
1800	5135	8168	13193	9516	6757	9751	14477	3255	8402	4358	3293	3330	3940	11108	8379	14245	28992	34913	44438	7902	37115	3470
1980	5627	8992	15638	10688	7461	11136	16594	3357	9657	4534	3356	3369	4280	12862	9845	16449	30890	38272	47464	8704	41919	3522
2160	6016	9672	18408	12302	8201	12812	19405	3258	11132	4876	3217	3489	4399	14706	11022	19112	32684	40917	48825	9556	45783	3640
2340	6314	10946	21504	13817	9075	14604	21876	3365	12782	5182	3392	3476	4563	16716	12394	21838	33792	42596	49001	10421	48882	3733
2520	6659	11806	24217	15144	9915	16249	24469	3343	14466	5116	3443	3551	4818	18599	14018	24695	34080	43112	48060	11267	51610	3648
2700	7353	12927	27417	16644	11067	18079	27215	3509	16212	5554	3391	3563	4985	20865	15278	27282	34591	43566	48250	12342	52983	3842
2880	7708	14098	30606	18419	11956	20201	29952	3479	18606	5878	3507	3717	5254	23723	17091	30126	35195	43580	46881	13118	54691	3915
3060	8518	15093	34165	20447	13221	22263	33545	3574	20637	6059	3467	3560	5543	25550	19127	33382	34674	43118	47047	14123	54102	3929
3240	8958	16584	37540	22234	14224	24120	35738	3717	23643	6534	3607	3670	5787	28103	20610	35827	34809	43282	46959	14584	55683	3841
3420	9492	18043	40183	23981	14992	26387	38927	3643	25721	6887	3756	3772	5890	30340	22465	38397	34194	43152	45986	15973	55666	4026

Table S10. Fluorescence -CRISPR Cas12a was used to detect fluorescence intensity in 62 samples.

Time (s)	A23	A24	A25	A26	A27	A28	A29	A30	A31	A32	A33	A34	A35	A36	A37	A38	A39	A40	A41	A42	A43	A44
0	5286	4612	4761	4417	3883	4005	3372	4251	3720	4624	4298	4280	3559	3244	3407	4040	2661	2655	2751	2708	2759	2512
180	5868	4938	4963	4661	3893	4168	3443	4506	3918	4791	4323	4394	3797	3464	3520	4545	2781	2765	2870	2851	2872	2633
360	6854	5261	5474	5146	3999	4417	3447	4649	3928	5273	4633	4820	4013	3424	3552	4752	2839	2686	2845	2821	2802	2607
540	8185	6280	6189	6054	3872	4875	3396	5357	3875	6213	4997	5227	4351	3600	3539	5370	2696	2710	2878	2879	2786	2744
720	10340	7818	7614	7918	3928	5900	3396	6917	3834	7681	5569	6224	4977	3687	3685	6072	2783	2795	2913	2856	2845	2696
900	13899	10292	9684	10736	3976	7864	3384	9551	4052	10109	6672	8014	5712	3881	3690	7177	2756	2845	2847	2868	2733	2661
1080	17742	14394	12927	14424	4121	10574	3313	13806	4370	14573	8695	10447	6730	4332	3840	8251	2809	2741	2934	2938	2838	2676
1260	23299	19523	17513	19508	4151	13374	3483	19083	4604	19910	10523	13535	7911	4578	3944	9589	2807	2823	3028	2837	2756	2746
1440	28727	25006	22623	25165	4287	17043	3361	24894	4918	25498	12831	16515	9277	4969	4384	10919	2769	2757	2932	2893	2831	2747
1620	33787	31278	28157	31281	4440	20168	3487	31086	5409	32298	15413	20021	11181	5735	4736	12326	2788	2796	2843	2871	2864	2707
1800	38579	37917	34626	36693	4549	23348	3433	36787	5637	38004	17878	23170	13195	6578	5199	14485	2817	2796	2941	2866	2869	2739
1980	43433	41947	39681	42627	4620	26598	3444	42050	6226	43604	20368	26592	15521	7387	5769	16140	2842	2799	2990	2830	2918	2770
2160	46929	46978	44527	47344	4697	29685	3562	46629	6650	48455	23036	28739	18113	8398	6650	18294	2865	2739	3057	2921	3031	2735
2340	48903	49606	49549	50902	4883	32262	3626	49293	7329	52947	24785	31469	21361	9806	7657	19755	2961	2839	3008	2934	2986	2709
2520	51149	52792	52991	53870	5115	34654	3697	51600	7757	56035	27537	34088	24801	10850	8827	22645	2965	2854	3088	2896	3135	2838
2700	52492	54347	54837	50109	5337	37243	3786	53628	8253	55373	28888	35897	28215	12161	10162	24399	3010	2898	2984	3008	3154	2875
2880	52372	54847	56180	55581	5600	38760	3879	55126	8740	57225	31104	37740	31760	14064	11718	26306	3072	3095	3060	2995	3200	3011
3060	53099	54939	57073	55194	5735	40658	4054	56147	9396	59661	32523	39239	35280	15835	13174	28037	3207	3022	3133	3134	3329	2984
3240	54080	55048	56589	56404	5973	42496	4095	56976	9961	60949	33752	40532	40219	17786	14528	29712	3144	3069	3197	3180	3497	3052
3420	54510	54521	57769	55282	6157	43347	4230	56722	10567	62337	35376	41910	43254	20303	16351	31542	3157	3164	3153	3147	3570	3060

Table S11 Fluorescence -CRISPR Cas12a was used to detect fluorescence intensity in 62 samples

Time (s)	A45	A46	A47	A48	A49	A50	A51	A52	A53	A54	A55	A56	A57	A58	A59	A60	A61	A62
0	2842	2636	2839	2700	2686	3566	3046	3079	3084	3082	2959	2570	2614	2946	3052	3451	3167	4401
180	2897	2625	2740	2764	2762	3673	3029	3264	3119	3140	3019	2644	2606	2991	3246	3458	3188	4442
360	2871	2713	2795	2843	2820	3608	3063	3230	3128	3206	2978	2690	2735	3060	3223	3542	3114	4584
540	2900	2716	2846	2815	2832	3602	3061	3254	3152	3214	3032	2683	2706	3067	3286	3406	3164	4841
720	2916	2620	2793	2766	2771	3803	3092	3294	3258	3228	3071	2719	2656	3081	3223	3646	3259	4685
900	2810	2761	2730	2901	2838	3742	3147	3304	3216	3306	3080	2771	2638	3056	3247	3658	3305	4785
1080	2879	2681	2829	2850	2818	3639	3178	3349	3202	3289	3118	2777	2782	3183	3341	3859	3373	4823
1260	2932	2731	2761	2821	2886	3658	3230	3401	3290	3424	3095	2760	2675	3342	3332	3835	3480	4844
1440	2841	2746	2793	2835	2837	3741	3380	3325	3390	3393	3173	2855	2758	3313	3359	3910	3356	4989
1620	2830	2752	2856	2754	2887	3777	3402	3359	3336	3499	3222	2816	2758	3450	3429	4076	3558	4886
1800	2965	2724	2798	2862	2883	4029	3593	3476	3346	3430	3307	2974	2787	3570	3385	4136	3556	5028
1980	2962	2731	2777	2888	2852	4011	3645	3626	3498	3514	3370	2920	2848	3608	3508	4280	3687	5072
2160	3027	2822	2860	2936	2895	4220	3739	3752	3494	3531	3438	3076	2856	3884	3621	4296	3884	5196
2340	3074	2893	2924	2977	2939	4233	3795	3777	3518	3657	3313	3117	2882	4076	3675	4574	3975	5209
2520	3072	2862	3014	2973	2948	4178	3951	3839	3651	3656	3496	3239	2965	4206	3798	4793	4066	5170
2700	3190	2912	2959	2963	2985	4370	4136	3997	3818	3790	3474	3217	3001	4253	3865	4918	4071	5352
2880	3307	2987	3033	3006	3072	4395	4157	3986	3716	3851	3593	3264	3109	4327	3775	5020	4406	5295
3060	3276	3074	3106	3026	3107	4686	4247	4096	3860	3898	3688	3346	3014	4675	4040	5344	4575	5489
3240	3317	3163	3092	3102	3159	4610	4496	4205	3920	3883	3747	3343	3080	4785	4081	5399	4663	5719
3420	3376	3186	2979	3173	3207	4772	4630	4311	4033	3992	3666	3451	3231	4942	4040	5654	4882	5720
